# Transcatheter Aortic Valve Implantation for Severe Chronic Aortic Regurgitation

**DOI:** 10.3390/jcm13102997

**Published:** 2024-05-20

**Authors:** Judy Al Ahmad, Edward Danson

**Affiliations:** Department of Cardiology, Wollongong Hospital, Wollongong, NSW 2500, Australia

**Keywords:** transcatheter aortic valve implantation, aortic regurgitation, bicuspid aortic valve, valve-in-valve TAVI, JenaValve^TM^

## Abstract

Transcatheter aortic valve implantation (TAVI) has revolutionised the management of aortic valve disease, offering a less invasive alternative to traditional surgical valve replacement for severe aortic stenosis (AS). TAVI for pure aortic regurgitation (AR) is less well established, and, in fact, it was previously labelled as a relative contraindication. However, TAVI has been utilised for selected cases of pure or predominant AR. The primary limitations regarding the use of TAVI in AR are related to the absence of anatomical factors seen in patients with AS that have contributed to the safe and stable functioning of current-generation prostheses. These include aortic root dilatation, mobile valve leaflets and labile blood pressure within the aortic root, which may further increase the risk of valve migration and periprosthetic leak after deployment. Furthermore, patients with AR have more heterogeneous aortic root anatomies when compared to the population of patients with calcific or degenerative AS. This review article describes the current evidence for the off-label use of TAVI in pure AR and the various clinical syndromes associated with AR where there may be specific challenges in the application of TAVI.

## 1. Introduction

TAVI for the treatment of severe symptomatic AS is now the dominant therapy for patients with this disease [1]. The first-generation TAVI devices faced challenges in terms of valve design, delivery systems and procedural techniques; however, subsequent generations have evolved to include significant improvements in these domains, enhancing the stability of the prosthesis at the level of the aortic annulus, the seal around the frame, as well as the delivery of the implant through lower profile and more reliable delivery systems.

**Figure 1:**
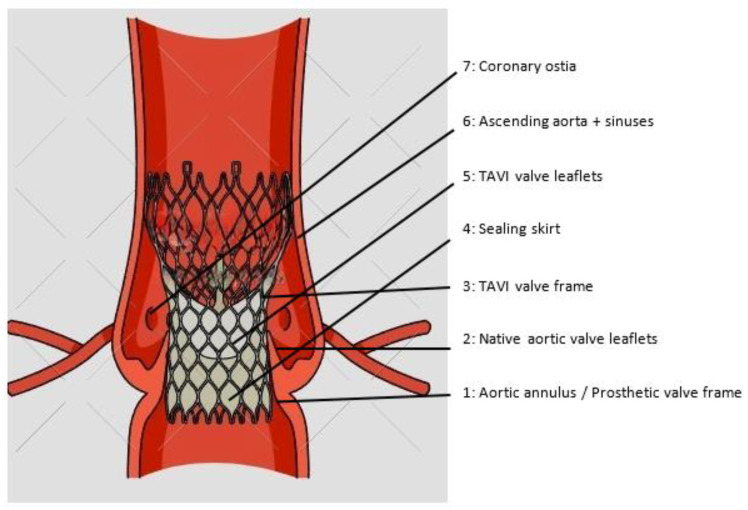
Anatomy of a TAVI.

**Figure 2:**
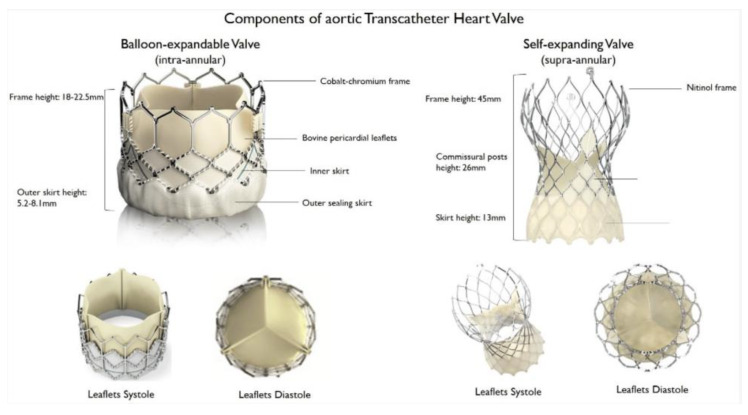
Anatomy of a balloon-expandable and self-expandable TAVI. Reprinted from Maurio Chiarito et al. [2].

TAVI in pure AR is not a first-line treatment, and the experience of this is relatively limited. The reason for this is largely because pure AR is seen in association with a much more heterogenous group of clinical syndromes; in addition, the anatomical factors seen in this situation are not necessarily suited to the design of the current generation of TAVI valves. The important anatomical considerations in this discussion are summarised in Figure 1, and these are referred to with respect to different patient subtypes throughout this review article. Firstly, native or bioprosthetic valve leaflets are relatively immobile in patients with severe AS and are typically thickened, which is a factor that helps the TAVI frame stabilise and ultimately anchor following deployment. In contrast, in patients with AR, the leaflets may be hypermobile, degenerated or friable, creating a somewhat unpredictable risk of valve or leaflet embolisation and interaction with the coronary artery ostia or sinuses. The native aortic annulus (prosthetic valve frame or, alternatively, neoannulus in patients with degenerated prostheses) may be significantly more heterogeneous in terms of shape and size in patients with AR, depending on the underlying cause. The Sinuses of Valsalva and ascending aorta are frequently enlarged or abnormal in patients with native AR, and they may be confined and hazardous in terms of proximity of the valve and coronary arteries in those with prosthetic AR. The sealing skirt of modern TAVI prostheses has been designed and marketed to minimise paravalvular leak in patients with calcific AS; however, this narrows the margin of error in depth of implantation. This may be harder to reliably achieve in patients with AR, particularly those with labile haemodynamics within the aortic root. Where the TAVI is implanted too deep, the sealing skirt may not have any impact on a paravalvular leak; however, if the TAVI is implanted too high, this may increase the risk of coronary obstruction. Finally, where severe AS is characterised by a single high-velocity forward jet, the haemodynamics within the aortic root in severe AR are more dynamic as a result of ventricularised pressure, alternating high-volume forward and backward flow and frequently eccentric regurgitant jets. With these factors to consider, stable implantation of a modern generation TAVI device is possible but likely to be less predictable. Figure 1 and Figure 2 demonstrate the anatomy and structure of different types of TAVI valves.

Over the last few years, new generation devices (such as the J valve^TM^ and Jena Valve^TM^) have shown promising results in regard to addressing these challenges and allowing for reasonable valve positioning and anchoring. To appropriately select patients, a detailed peri-operative assessment is necessary, which includes a multidisciplinary heart team assessment and a comprehensive 3D assessment of the aortic valve through transoesophageal echocardiography (TOE) and computed tomography (CT) to gain detailed information about the aortic valve annulus, leaflets and aortic root, allowing for appropriate valve sizing. A multidetector CT also provides crucial information about a patient’s iliofemoral anatomy for vascular access. A review of the latest case series and registry data has shown success rates between 75–100% [3,4,5,6,7,8,9,10]. Table 1 highlights several case series and registries for TAVI in AR based on the type of device, with newer generation devices demonstrating better outcomes.

It is important to note that there have been no randomised control trials investigating the use of TAVI in severe AR, highlighting that current available evidence stems largely from observational studies. Despite their inherent observational nature, these studies have demonstrated reasonable outcomes in patients with prohibitive surgical risks. Such patients would otherwise be managed with optimal guideline-directed medical therapy only, which typically results in substantial morbidity and mortality [17]. Most recently, the ALIGN-AR trial, a single-arm multicentre prospective trial, has shown a 92.8% procedural success, with significant improvement being seen in left ventricular end systolic diameters and quality of life (as measured by KCCQ-OS and the New York Heart Association (NYHA) functional class) [18].

## 2. Patient-Specific Considerations in the Application of TAVI for Severe AR

### 2.1. Pathophysiology of AR and Indications for Intervention

The pathophysiology of AR is heterogeneous across a spectrum of diverse groups of patients, often with a different primary pathology. Broadly, most patients will have diseased or degenerative aortic valve leaflets or an aortic root anatomy structurally related to the valve. This can result from congenital or acquired valvular disease and dilatation or deformation of the aorta itself. The most common congenital anomaly is a bicuspid aortic valve. Acquired causes often involve degenerative changes, such as aortic valve calcification or rheumatic heart disease. Other causes include infective endocarditis and connective tissue disorders. The regurgitant flow in severe AR leads to volume overload of the left ventricle, increased wall stress and subsequent ventricular remodelling. Acute AR is commonly caused by infective endocarditis, aortic dissections or bioprosthetic structural valve deterioration, which can be because of an acute leaflet tear.

Aortic regurgitation affects around 13% of the population; however, severe aortic regurgitation has a relatively low prevalence, estimated to be around 0.5%−2% [19]. Prevalence increases with age, particularly in men, although it is important to note that patients with AR are younger than those with AS, necessitating a durable intervention. This is contrary to patients with severe AS, who are often elderly, frail and may have prohibitive surgical risks, elements which would make them seek a minimally invasive procedure that offers symptomatic relief and quality of life rather than a durable long-term solution to their disease.

The clinical presentation of AR can vary depending on the severity and chronicity of the condition. Acute aortic regurgitation often requires urgent surgery due to rapid cardiac decompensation. It can occur as a result of aortic dissection, infective endocarditis, trauma or can even be iatrogenic due to a catheter-based cardiac procedure. On the other hand, patients with chronic aortic regurgitation may remain asymptomatic for a long period. Symptoms typically manifest as palpitations or heart failure symptoms, including exertional dyspnoea, orthopnoea, fatigue and peripheral oedema. Severe AR is characterised by an effective regurgitation orifice area >0.3 cm^2^, regurgitant volume >60 mL, a regurgitant fraction of >50% and a Left ventricular outflow tract (LVOT) vena contracta of >0.6 cm, as well as a pressure half time of <200 ms. The gold standard treatment of severe symptomatic AR is surgical aortic valve replacement (SAVR) regardless of left ventricular function. SAVR is also indicated in patients with asymptomatic severe AR and left ventricular dysfunction with an ejection fraction of less than 55% or a LVESD of >50 mm or LVESD >25 mm/m^2^. SAVR is also indicated in patients with severe asymptomatic AR undergoing CABG or surgery on another cardiac valve. [17]. Without intervention, mortality can be as high as 20% [20,21]. Thourani VH et al. found that there was a 62% reduction in mortality in patients with severe symptomatic AR who underwent SAVR compared to those who did not undergo surgery [22]. This highlights the need for alternative minimally invasive therapy to reduce morbidity and mortality in these patients with prohibitive surgical risk.

### 2.2. Subtypes of AR

#### 2.2.1. AR in Degenerative Trileaflet Disease without Aortopathy

AR in patients with degenerative aortic valve disease without a congenital substrate might be apparent because of myxomatous disease, prior endocarditis [23] or asymmetric degenerative calcific disease. In these patients, the major challenge to offering TAVI in a patient unsuited to a surgical option relates to overcoming the consequences of thin or hypermobile leaflets (which do not facilitate device-anchoring or stability during deployment) and the haemodynamic environment within the aortic root. Whilst one may hypothesise that this might be best overcome by an oversized self-expanding/repositionable prosthesis, a study of patients undergoing TAVI for pure AR without aortopathy revealed that the newer generation of balloon expandable (BE) valves performed the best with respect to short-term clinical outcomes with less oversizing required [14]. Furthermore, it would seem from this study that the extent of residual AR following TAVI in this patient group was independently associated with adverse outcomes, which were relatively frequent in the group as a whole.

#### 2.2.2. AR Associated with Aortopathy

**Figure 3:**
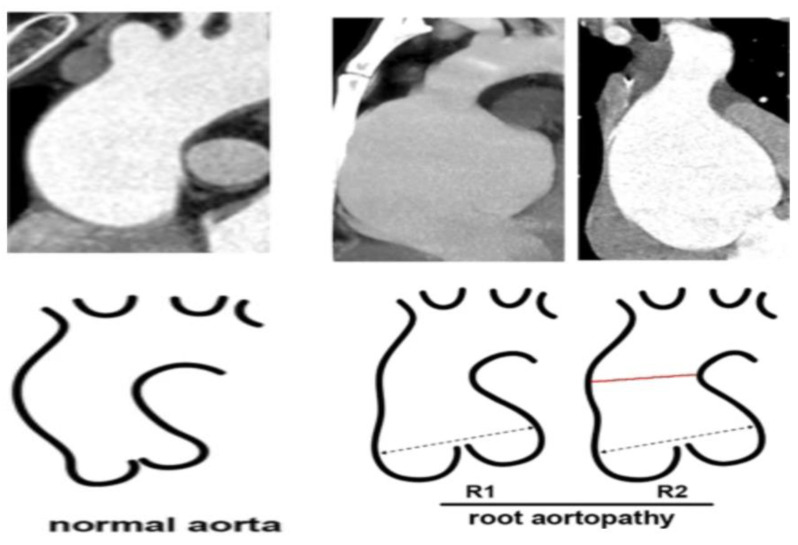
Aortopathy classification highlighting limitations in the use of TAVR with deformed and enlarged anatomy. Dotted line represents the widest part of the aorta. R1 depicts aortic root dilatation without ascending aorta dilatation. R2 depicts aortic root dilatation with the red line demonstrating ascending aorta dilatation. (Modified and reprinted from Minhjia Ma et al. [25]).

AR due to aortopathy results from noncoaptation of the aortic leaflets secondary to aortic root dilatation. This can be congenital (i.e., due to various types of connective tissue disorders such as Marfan syndrome, Ehlers−Danlos syndrome and Turner syndrome) or acquired (i.e., ascending aortic dilation or aneurysm formation due to long-standing hypertension and atherosclerosis). An aortic root >5.5 cm increases the risk of aortic dissection and consequential mortality. The treatment of AR secondary to aortopathy can be particularly challenging, as the annular diameters can quickly change if the procedure is not performed promptly after imaging is obtained. Figure 3 highlights a few types of root aortopathy. Furthermore, significant aortic root dilatation necessitates a replacement of the ascending aorta to mitigate risks of aortic dissection, a procedure which would be best performed alongside a SAVR. There is no published data on the use of TAVI for the treatment of aortopathy-related aortic regurgitation However, the progression of aortopathy associated with aortic stenosis has been shown to be reduced by TAVI [3]. It is also worth noting that several less surgically intensive strategies exist to stabilise aortic root dilatation in those who do not require full aortic root replacement (including external aortic wrapping [24] or the PEARS (Personalised external aortic root support) procedure, both of which can be performed off-pump [3]; additionally, TAVI could be used in conjunction with minimally invasive surgical strategies such as these in theory.

#### 2.2.3. AR Associated with Bicuspid Aortic Valve

**Figure 4:**
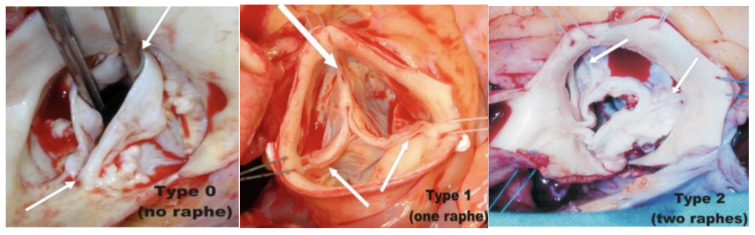
The Sievers and Schmidtke classification system for the bicuspid aortic valve. Type 0 BAV with arrows highlighting the 2 commissures. Type 1 BAV with large arrow highlighting the raphe between the fused left and right coronary cusps and small arrow depicting two completely developed commissures. Type 2 BAV with arrows depicting the two raphes and high grade stenosis. (reprinted from The Journal of Thoracic and Cardiovascular Surgery, 133, H.-H. Sievers and C. Schmidtke, A classification system for the bicuspid aortic valve from 304 surgical specimens, 1226–1233, © 2007) [30].

Bicuspid aortic valve (BAV) disease is the most common congenital heart defects, occurring in 1–2% of the population and resulting in aortic valve stenosis, regurgitation or a combination of both [26]. Several phenotypes are known and classified variously according to the Sievers or Dichotomous classification [27]. Figure 4 highlights the types of bicuspid aortic valves as classified by the Sievers and Schimdtke classification system. There is now comprehensive experience of TAVI for patients with severe aortic stenosis and bicuspid anatomies [28]. However, anatomical challenges exist in these patients, particularly those managing abnormally low calcium burdens, elliptical, shallow or dilated anatomies, as well as those associated with predominant AR. Certain valves, such as the J-valve^TM^, are specifically designed for a trileaflet valve with three anchoring rings; this would result in technical challenges in deployment in a bicuspid aortic valve, particularly in bicommisural non-raphe- type BAVs (Sievers 0) [29]. Although there are various case reports and a series on the use of TAVI for pure AR, no detailed subgroup analyses have shown the inclusion of patients with bicuspid-related AR. Data is thus scarce, and there are no reports on the use of TAVI in pure or predominant AR associated with bicuspid aortic valves. In a single centre prospective study, Jung J et al. found that patients with bicuspid aortic valve stenosis, and an ascending aorta of <50 mm, who underwent TAVI did not experience progressive aortopathy over a 12-month follow-up period (aortic diameter change −0.11 mm + −1.9 mm/year SD *p* = 0.50) [3]. Further large-scale studies are necessary to support these findings.

BAVs more commonly have an elliptical annulus and/or heavy asymmetric calcification. In patients with severe AS, this can sometimes be overcome by achieving higher implantation of the transcatheter heart valve at the narrowest part of the commissure, although this may increase the risk of a paravalvular leak, which is a major downside in a patient with predominant AR. The varying raphe anatomy and aortic root dilatation in patients with BAVs may also increase the risk of coronary occlusion, aortic annulus rupture and heart block after TAVI due to interaction with the posterior non-coronary cusp, which lies close to the atrioventricular node [4,5].

#### 2.2.4. AR associated with Bioprosthetic Aortic Valve Replacement

Bioprosthetic aortic valve replacement has been increasingly favoured, particularly in elderly patients, due to the lack of reliance on long-term anticoagulation and complications associated with anticoagulation. However, it is important to acknowledge that bioprosthetic valves are less durable, with an average lifespan of 10–20 years. Bioprosthetic valve failure can lead to valve stenosis, regurgitation, or a combined pathology. However, presentation with acute AR is relatively common and associated with significant morbidity and mortality [6]. Over the last decade, valve-in-valve TAVI has emerged as an alternative, lower-risk treatment option in regard to re-performing open heart surgery in patients who are often acutely unwell with multiorgan dysfunction. The PARTNER-2 valve-in-valve registry demonstrated good functional outcomes in regard to the use of TAVI in failing bioprosthetic valves in high-risk patients with prohibitive surgical risks, featuring a low risk of procedure-related morbidity and mortality [7]. In comparison to TAVI for native pure AR, valve-in-valve TAVI for AR secondary to failing bioprostheses had a lower risk of mortality and a more significant improvement in NYHA functional status [8]. The anatomical considerations in offering TAVI in the case of failing aortic bioprostheses are critical and should not be abbreviated despite the acuity of some patients with this disease.

The relationship between a failing prosthetic valve and the native annulus, sinus and coronary anatomy has important implications regarding coronary artery perfusion and the valve haemodynamics following TAVI to correct the condition. To reduce the risk of interaction of the bioprosthetic aortic leaflets and the coronary circulation, several techniques have been developed. The BASILICA technique (bioprosthetic or native aortic scallop intentional laceration to prevent iatrogenic coronary artery obstruction during TAVI) is a transcatheter technique based on the intentional laceration of the anterior mitral leaflet to prevent left ventricular outflow obstruction (LAMPOON) technique. BASILICA is an off-label technique which uses electrocautery to lacerate the leaflet considered most likely to affect coronary perfusion through sinus sequestration or direct leaflet interaction with the coronary ostium once pinned by a TAVI valve. The technique aims to divide the leaflet into two segments to allow for perfusion through the acquired defect. Another technique, Bioprosthetic Valve Fracture, utilises a noncompliant balloon to fracture the suture ring of the bioprosthetic valve in order to reduce patient−prosthesis mismatch, which will have an important impact on the valve haemodynamics and durability [9]. Finally, the Chimney−snorkel technique involves the deployment of a stent from the coronary artery at risk to above the bioprosthetic leaflet to achieve perfusion into the coronary from behind the pinned bioprosthetic leaflet following TAVI [10]. Whilst these adjunct techniques are in widespread use globally and are included in large registries, they are inevitably associated with increased risks, and re-performed surgery may be a lower-risk option in some patients.

### 2.3. Limitations for Intervention in AR

**Figure 5:**
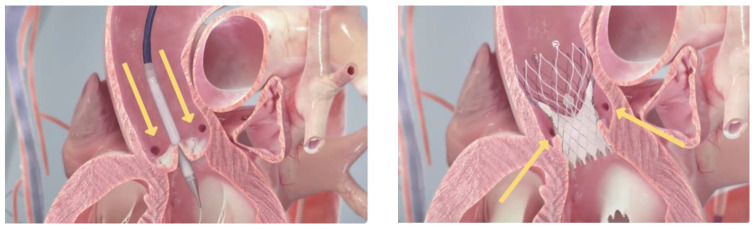
Transcatheter aortic valve implantation—yellow arrows highlighting coronary sinuses.

The use of TAVI in severe native AR poses significant challenges due to the underlying anatomy. As highlighted above, unlike aortic stenosis, the pathology leading to AR is not limited to defects in the aortic leaflets; rather, it involves clinically diverse pathologies, making it difficult to design a valve that fits all. Perioperative planning for valve selection includes a multidetector CT, transthoracic echocardiography, and sometimes transoesophageal echocardiography. It is crucial to obtain accurate dimensions of the annulus, ascending aorta, Sinuses of Valsalva, the sinotubular junction and any prosthetic material. A dilated elliptical annulus in cases with severe AR makes it very difficult to size valves appropriately, raising the possibility of residual paravalvular leakage. While valve over-sizing and BE devices may help overcome sizing and seal challenges, this remains difficult and increases the risk of annular rupture, valve migration and permanent pacemaker implantation due to conduction issues. Often there is a lack of leaflet calcification, which usually serves as an anchor and fluoroscopic guide for valve implantation. This increases the risk of prosthesis migration and risk of procedure failure. In native pure AR, there may be poor visibility on fluoroscopy due to contrast washout from severe regurgitation, and the elevated stroke volume can lead to increased risk of transcatheter valve embolisation. The dilated thinned ventricular wall associated with progressive AR may also lead to an increased risk of wire perforation [11,12,13,14,15,31]. Figure 5 shows the process of implantation of a TAVI.

In a multicentre registry of 331 high-risk surgical patients using early and new-generation valves for severe AR, Yoon et al. found that patients with minimal aortic valve calcification had reduced device implantation success using early generation valves. However, procedure outcomes were not dependent on aortic valve calcification when new-generation valves were used [14]. Valve oversizing >15% was linked to improved post-procedural outcomes, yet there is no consensus data on the best method for appropriate sizing, and over-sizing >20% can also be associated with significant complications, particularly annular rupture and device migration [14].

### 2.4. TAVI in AR—Safety and Efficacy

To date, there have been no randomised clinical trials which support the use of TAVI in AR; however, several small-scale case series and registries have evaluated the efficacy and safety of TAVI in AR [9,10,11,12,13,14,15,31]. Roy et al. assessed the use of the CoreValve^TM^ (first-generation valve) in severe AR in 43 patients. Successful implantation occurred in 97.7%; however, 18.6% required a second valve in valve implantation due to significant residual AR, while a large proportion of patients (79%) had Grade 1 residual AR. Notably, most patients (62%) had AR because of leaflet degeneration with no aortic root dilatation [8]. Although this was a small single-centre retrospective registry, it demonstrated significant improvement in the functional NYHA class of patients undergoing TAVI for AR.

The PANTHEON (Performance of Currently Available Transcatheter Aortic Valve Platforms in Inoperable Patients with Pure Aortic Regurgitation of a Native Valve) study is a multicentre retrospective study that evaluated the use of self-expandable (SE) and BE transcatheter heart valves for severe native pure AR in 201 patients amongst 16 centres in Europe and the USA. Overall, there were no significant differences between SE and BE devices in terms of technical and device success rates, which were overall 83.6% and 76.1%. The rate of composite endpoint at 1 year (all-cause mortality and heart failure hospitalisation) was high (25.7%). The incidence of transcatheter valve embolisation and migration (TVEM) occurred in 12.4% of patients, with the main causes being malpositioning, over-expansion > 20%, post dilatation and transcatheter heart valve failure to anchor due to a lack of calcification. Interestingly patients undergoing TAVI for AR had fewer complications of stroke and more complications necessitating permanent pacemaker implantation. While lack of leaflet calcification may reduce the risk of calcium embolisation and stroke, it also renders the atrioventricular node more susceptible to defacement. The PANTHEON study therefore establishes the feasibility of using current-generation TAVI valves for AR, acknowledging several limitations in the context of certain typical anatomical features in this patient group and highlighting the need for dedicated AR-TAVI devices [32].

**Figure 6:**
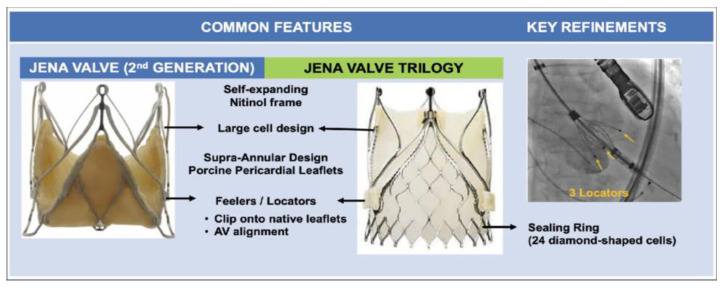
The JenaValve Trilogy (reprinted from Zaid et al. [36]).

At present, the JenaValve^TM^ and J valve^TM^ (JieCheng Medical Technology Co., Ltd., Suzhou, China) are the only devices that are approved by the CE (European Conformity) and the China Food and Drug Administration for the treatment of pure AR, respectively [33]. The JenaValve^TM^ is delivered transfemorally. The SE nitinol stent and anchor rings that clip onto the native aortic valve leaflets allow for appropriate anchoring in the absence of aortic valve calcification [34] as shown in Figure 6. The J valve^TM^ has a three-point clasper connected by three sutures, making the valve movable across the clasper to optimise positioning. The clasper also serves to clip the native leaflets [35].

In a German multicentre case series of 31 patients who underwent JenaValve^TM^ transapical implantation, Seiffert et al. showed a 96.8% successful valve implantation rate, and only 9.7% of patients had mild residual AR, with no patients having residual moderate or severe AR. Despite these results, 30-day mortality and 6-month mortality were high at 12.9% and 19.3%, respectively. However, in this case series, the JenaValve^TM^ was implanted via a transapical approach, which may have led to an increased risk of operative recovery, stroke and mortality due to the complexity of the procedure [16].

The JUPITER registry, another European registry, of 30 patients that underwent TAVI for AR using transapical implantation of the JenaValve^TM^, demonstrated a comparable procedural success rate at 96.7%, with mild to trivial residual AR at 30 days (84.6%) and only one patient requiring open surgical bailout. Survival at 1 year was 79.9%, and there was a low pacemaker implantation rate of 3.8% [37].

Similar results were shown at 30-days post-device-implantation by Adam et al. in 2022 in a multicentre German trial of 56 patients with severe AR and NYHA III-IV where 98% of JenaValve^TM^ devices were successfully deployed and featured no moderate or severe residual AR and no valve embolisation. In this study, there was a significantly lower 30-day mortality rate (1.7%); this could be attributed to access site (transfemoral) as well as operator skill and experience [38].

The safety of TAVI in AR was highlighted in a large systematic review by Yousef et al. where they found a comparable 30-day and 1-year mortality rate in patients undergoing TAVI for AR vs. TAVI for AS. The 30-day mortality in patients undergoing TAVI for AR was also similar to patients with prohibitive surgical risks undergoing SAVR, emphasizing that TAVI in AR appears safe and comparable in the immediate setting and short term follow up [39]. 

At present the largest prospective single-arm, multicentre trial, The JenaValve^TM^ ALIGN-AR Pivotal trial, has set out to examine the long-term safety and efficacy of the JenaValve^TM^ Trilogy^TM^ transcatheter heart valve in the treatment of severe symptomatic AR over 5 years. An amount of 180 patients with prohibitive surgical risks, as determined by a heart team, and with an NYHA of at least II were recruited. A procedural success rate of 92.8% was achieved with no intra-operative deaths. The primary safety endpoint was a composite at 30 days of all-cause mortality, major bleeding, stroke, acute kidney injury, new pacemaker implantation or valve dysfunction requiring surgical or percutaneous intervention. The primary efficacy endpoint was all-cause mortality at 12 months. The primary safety endpoint occurred in 26.7% of patients at 30 days, while the primary efficacy endpoint occurred in 7.8% (*p* < 0.0001). Both values met the non-inferiority pre-set criteria. The rate of post-procedure pacemaker implantation was high (24%). However, lower rates were observed nearing the end of the trial, which could be attributed to a refinement in insertion technique, advancements in the management of peri-procedural conduction issues and a reduction in oversizing. At 12 months, echocardiography findings demonstrated significant LVESD improvement from an average of 39.6% to 34.2% (*p* < 0.0001). There was a significant improvement in patients’ NYHA functional class and quality of life, with most patients exhibiting NYHA I symptoms and no patients experiencing NYHA IV symptoms at 12 months [18].

## 3. Conclusions

Whilst it is an encouraging landscape for development as a therapy, the evidence base for TAVI in AR is relatively limited compared to AS. Registries and case reports provide valuable real-world data that support the use of TAVI in selected patients with AR where the anatomical substrates are considered very carefully with respect to the patient and a TAVI prosthesis being used. Randomised controlled trials and larger prospective studies specifically focusing on TAVI for AR are still needed to establish its efficacy, durability, and long-term outcomes in this patient population. Second-generation valves, particularly the JenaValve^TM^ and J-valve^TM^, have shown favourable results. ALIGN-AR is the first prospective interventional study, and, although it is single arm and non-randomised, it has shown encouraging results at 12 months. Follow-up results at 5 years will be crucial to determine the long-term safety and efficacy of the JenaValve^TM^. ALIGN-AR will hopefully serve as a landmark study to pave the way for future randomised clinical trials and FDA approval, expanding the minimally invasive treatment for those patients with excessive surgical risk, particularly in an era featuring an increasingly frail population. Once further long-term data is established for TAVI in severe AR in high-risk patients, further research is warranted comparing SAVR to TAVI in patients with low to intermediate surgical risk.

## Figures and Tables

**Table 1:** Device type, patient characteristics, echocardiographic parameters, and success rates of included case series.

Study	Number	Device Type	Age	Mean LVEDD, mm	Success %
Sawaya et al. [8]	78	CoreValve^TM^, JenaValve^TM^, DirectFlow^TM^, Sapien XT^TM^, Sapien 3^TM^, Evolut R^TM^, Lotus^TM^	74	58.5	72%
Liu H et al. [11]	47	J-Valve^TM^	73.7	59.2	100%
Schofer et al. [12]	11	Direct flow valve^TM^	75	59	91%
Roy et al. [13]	43	CoreValve^TM^	75	59	98%
Yoon et al. [14]	331	CoreValve^TM^, JenaValve^TM^, DirectFlow^TM^, Sapien XT^TM^, Sapien 3^TM^, Evolut R^TM^, Lotus^TM^, Portico^TM^, Engager^TM^, Acurate^TM^	74	N/A	74%
Liu H et al. [15]	43	J-Valve^TM^	73.9	60.5	97.7%
Seiffert et al. [16]	26	JenaValve^TM^	73	N/A	97%
